# Thermally Tunable Bi-Functional Metasurface Based on InSb for Terahertz Applications

**DOI:** 10.3390/ma18122847

**Published:** 2025-06-17

**Authors:** Rafael Charca-Benavente, Rupesh Kumar, Ruth Rubio-Noriega, Mark Clemente-Arenas

**Affiliations:** 1 Graduate School, Universidad Nacional de Ingeniería, Lima 15333, Peru; juan.charca.b@uni.pe; 2 Wireless Sensing and Imaging Laboratory & 6G Research Laboratory, SRM University AP, Amaravati 522502, India; rupesh.k@srmap.edu.in; 3 Department of Engineering, Pontificia Universidad Catolica del Peru, Lima 15087, Peru; rrubion@pucp.edu.pe; 4 Electronics Circuits and Systems Research Group gECS-HF, Universidad Nacional Tecnológica de Lima Sur UNTELS, Villa El Salvador, Lima 15834, Peru

**Keywords:** metasurface, indium antimonide (InSb), terahertz (THz), polarization converter, perfect absorber, thermal tuning

## Abstract

In this work, we propose and analyze a thermally tunable metasurface based on indium antimonide (InSb), designed to operate in the terahertz (THz) frequency range. The metasurface exhibits dual functionalities: single-band perfect absorption and efficient polarization conversion, enabled by the temperature-dependent permittivity of InSb. At approximately 280 K, InSb transitions into a metallic state, enabling the metasurface to achieve near-unity absorptance (100%) at 0.408 THz under normal incidence, independent of polarization. Conversely, when InSb behaves as a dielectric at 200 K, the metasurface operates as an efficient polarization converter. By exploiting structural anisotropy, it achieves a polarization conversion ratio exceeding 85% over the frequency range from 0.56 to 0.93 THz, while maintaining stable performance for incident angles up to 45°. Parametric analyses show that the resonance frequency and absorption intensity can be effectively tuned by varying the InSb square size and the silica (SiO_2_) layer thickness, achieving maximum absorptance at a SiO_2_ thickness of 16 μm. The proposed tunable metasurface offers significant potential for applications in THz sensing, imaging, filtering, and wavefront engineering.

## 1. Introduction

In the last decade, the terahertz (THz) frequency band has emerged as a highly interesting research area due to its potential to significantly improve technologies such as communications [1], imaging [2], and sensing technologies [3,4]. This spectrum is positioned between the infrared and microwave regions of the electromagnetic spectrum; the THz band offers unprecedented bandwidth, making it an ideal candidate for high-speed, high-capacity communication applications [5]. For example, sixth-generation mobile communications are evaluating the use of the [0.1–10] THz range [6]. However, the development of efficient and versatile devices in this band has faced significant challenges, primarily due to the lack of materials and structures capable of effectively manipulating THz waves [7].

Metasurfaces are defined as artificial structures composed of subwavelength elements [8,9]. These structures have emerged as a promising solution to overcome these limitations. These structures enable unprecedented control over the electromagnetic properties of light, including its phase, amplitude, and polarization [10,11]. In particular, tunable metasurfaces, whose properties can be dynamically adjusted, offer the ability to adapt to different operating conditions and functionalities, making them especially attractive for applications in the THz band [12,13].

Indium antimonide (InSb) is characterized by its high electron mobility and an electric permittivity that varies significantly with temperature, making it an ideal candidate for designing devices with tunable electromagnetic properties [14]. At low temperatures, InSb exhibits typical semiconductor behavior, but as the temperature increases, its permittivity undergoes drastic changes, enabling efficient tuning of the optical response of metasurfaces [15]. This unique property has been exploited in the design of structures capable of dynamically modulating the phase, amplitude, and polarization of the THz waves [16]. Additionally, InSb is compatible with standard fabrication techniques, facilitating its integration into practical devices [17]. Its ability to respond to thermal stimuli makes it particularly attractive for applications in environments where thermal control is feasible [18], such as wireless communications systems, high-precision sensors, and medical imaging systems.

Other materials, such as vanadium dioxide (VO_2_), have received much more attention for the development of bi- or tri-functional devices [19]. VO_2_ offers binary switching via a first-order insulator–metal transition at ∼340 K, but this change carries a 3–10 K thermal hysteresis and extra THz optical loss [20]. By contrast, InSb is a narrow-gap semiconductor whose Drude permittivity shifts continuously with temperature, giving analog tunability without any structural phase change [21]. Its very high room-temperature electron mobility ($\mu_{e}\approx 7\times 10^{4}$ cm^2^V^−1^s^−1^) keeps the damping constant below 0.1 THz, so Joule losses are markedly lower than in VO_2_ [22].

Metasurfaces have been used to design polarization converters, enabling the efficient transformation of linear polarization into circular or elliptical polarization, and vice versa, through the design of their resonant elements [23]. This capability is particularly significant in applications such as wireless communications [24], where the manipulation of polarization can enhance spectral efficiency and increase data transmission capacity.

Moreover, metasurface-based absorbers have been designed to optimize THz wave absorption within specific frequency bands [25], which is crucial for applications in sensing, medical imaging, and camouflage systems [26]. These structures achieve near-perfect absorption by coupling electromagnetic resonances with the material properties, allowing precise control over the spectral response [27,28]. For example, a metal–dielectric metasurface using frequency-selective ring resonators has been shown to function as a variable attenuator in the 2–5 THz range, a band of interest for LIDAR and defense-related applications [29]. This further highlights the versatility of metasurfaces in tailoring THz functionalities for diverse practical uses.

The combination of absorption and polarization conversion within a single metasurface unlocks multifunctional capabilities for advanced THz applications. In wireless communication systems, polarization conversion improves spectral efficiency via polarization-division multiplexing, while resonant absorption can suppress back-reflections and ambient interference [30]. In sensing and imaging, tunable absorbers allow frequency-selective detection of analytes or materials, and polarization control enhances contrast or enables characterization of anisotropic samples [31,32]. Such reconfigurable metasurfaces offer compact, adaptable platforms capable of switching functionalities in real time.

The incorporation of phase-change materials, such as InSb, into metasurfaces remains largely unexplored. This limited exploration of phase-change materials in metasurfaces represents a missed opportunity, because these materials could enable adaptive systems capable of adjusting their functionality in real time. In light of this, this work aims to develop a bi-functional device that operates as both a polarization converter and a THz band absorber, leveraging the unique properties of InSb. Building upon our preliminary conference report [33], which presented an initial version of the device with a limited parametric study, the present work introduces a significantly improved design. This includes an enhanced metasurface layout for better dual functionality and a more comprehensive analysis of its performance under various polarization states, incidence angles, and thermal conditions.

This document is organized as follows. Section 1 introduces the motivation for tunable terahertz metasurfaces and the use of temperature-responsive InSb for dual functionality. Section 2 describes the metasurface design, including geometry, material modeling, and simulation setup. Section 3 presents the numerical results, demonstrating absorption, polarization conversion, and robustness to thermal and angular variations, along with a comparison to recent works. Section 4 concludes by summarizing the main findings and potential THz applications of the proposed metasurface.

## 2. Structure Design

### 2.1. Meta-Atom Description

**Figure 1:**
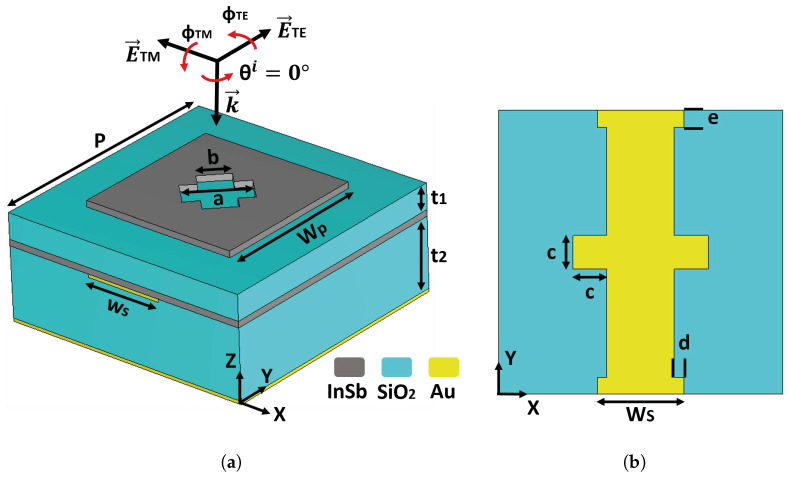
Geometry of the tunable THz metasurface. (**a**) Perspective view of one unit cell under normal incidence ($\theta_{i}=0^{\circ }$), (**b**) Top view of the embedded gold resonator. The angles $\varphi_{\mathrm{TE}}$ and $\varphi_{\mathrm{TM}}$ respectively define the polarization orientation of the incident wave for TE and TM configurations.

The meta-atom depicted in Figure 1 consists of six layers (top to bottom): (i) a square InSb patch with a cross-shaped aperture, (ii) a layer of silica (SiO_2_), (iii) a thin layer of InSb, (iv) a patterned gold (Au) strip on (v) a SiO_2_ substrate, and (vi) a thin Au backplane.

The meta-atom is square and bi-periodic in the *x*-*y* plane; its period is given by P= 160 μm. The InSb patch is also a square with $W_{p}=$ 100 μm. Within the patch, the aperture is given by a= 40 μm and b= 20 μm. The Au strip pattern in Figure 1 (right) is characterized by $W_{s}=$ 48.86 μm, c= 19 μm, d= 5.43 μm, e= 9.5 μm and is 2 μm thick. Lastly, SiO_2_ layers are $t_{1}=$ 16 μm and $t_{2}=$ 44 μm, while each InSb film is 4 μm thick.

For practical fabrication, the thin InSb film can be deposited using Molecular Beam Epitaxy (MBE) or Chemical Vapor Deposition (CVD), both of which offer precise control over thickness and uniformity [34,35,36].

### 2.2. Material Description

The dielectric permittivity of indium antimonide (InSb) is modeled using the hybrid Drude model [37]:

(1) $$\varepsilon_{\mathrm{InSb}}=\varepsilon_{\infty }-\frac{\omega_{p}^{2}}{\omega^{2}+i\gamma \omega }$$

where $\varepsilon_{\infty }=15.68$ is the high-frequency relative permittivity, and $\gamma =\pi \times 10^{11}$ rad/s is the damping constant. Since γ is inversely proportional to the electron mobility μ as $\gamma =em^{*}/\mu$, it is expected to vary with temperature. However, the mobility μ of InSb changes slightly from 160 K to 350 K in the 0.1–2.2 THz range [38,39,40,41]. Therefore, it is reasonable to approximate γ as constant for modeling purposes. For example, InSb has an electron mobility of $77,000\;{\mathrm{cm}}^{2}/V\;\cdot \;$ s at 300 K, it implies rapid charge-carrier motion, which is critical for fast switching [42,43]. The term $\omega_{p}$ represents the plasma frequency, which depends on the intrinsic carrier density and is given by

(2) $$\omega_{p}=\sqrt{\frac{Ne^{2}}{0.015\varepsilon_{0}m_{e}}}$$

where $e=-1.6\times 10^{-19}$ C is the elementary charge, $m_{e}=9.11\times 10^{-31}$ kg is the electron mass, and $\varepsilon_{0}$ is the permittivity of free space. The intrinsic carrier density *N* is temperature dependent and follows the relation:

(3) $$N=5.76\times 10^{20}T^{1.5}\exp\left(-\frac{E_{g}}{2K_{B}T}\right)$$

where $E_{g}=0.26$ eV is the band gap energy, and $K_{B}=8.62\times 10^{-5}$ eV/K is the Boltzmann constant.

According to [44], at T=200 K, InSb is a dielectric. However, at 280 K, InSb transitions to the metallic state. Experimentally, precise and stable temperature control within [200, 280] K can be achieved using a continuously variable cryostat [45]. The electrical conductivity of Au in the terahertz regime is temperature-dependent, being approximately $6.84\times 10^{7}\;S/m$ at 200 K and $4.876\times 10^{7}\;S/m$ at 280 K [46], and the relative dielectric permittivity of SiO_2_ is 3.8 [47].

All electromagnetic simulations were carried out in CST Studio Suite (version 2025), a commercially available finite-integration-technique (FIT) solver. We employed the frequency-domain 3-D FIT engine with hexahedral meshing, periodic boundaries in the x and y directions, and open boundaries along z.

## 3. Numerical Results

### 3.1. Metasurface Single–Band Absorber

When InSb transitions into its metallic state at T=280K, the proposed metasurface behaves as a single-band absorber within the terahertz regime.

The absorptance of the metasurface can be obtained from the standard relation A=1−R−T, where $R=|S_{11}{|}^{2}$ is the reflectance and $T=|S_{21}{|}^{2}$ is the transmittance. Because the thickness of the 4 μm InSb film exceeds the penetration depth of THz radiation, the transmittance is negligible (T≈0). Hence, the absorptance simplifies to [19,48]:

(4) $$A=1-R=1-|S_{11}{|}^{2}$$

Impedance-matching description: Using impedance matching theory, the absorption can also be expressed in terms of the effective surface impedance *Z* of the metasurface and the free–space impedance $Z_{\mathrm{air}}$.

(5) $$A=1-R=1-{\left|\frac{Z-Z_{\mathrm{air}}}{Z+Z_{\mathrm{air}}}\right|}^{2}\;=\;1-{\left|\frac{Z_{r}-1}{Z_{r}+1}\right|}^{2}$$

where $Z_{r}=Z/Z_{\mathrm{air}}$ is the relative surface impedance. The latter is retrieved from the simulated *S*-parameters by [49]:

(6) $$Z_{r}=\sqrt{\frac{{\left(1+S_{11}\right)}^{2}-S_{21}^{2}}{{\left(1-S_{11}\right)}^{2}-S_{21}^{2}}}$$

**Figure 2:**
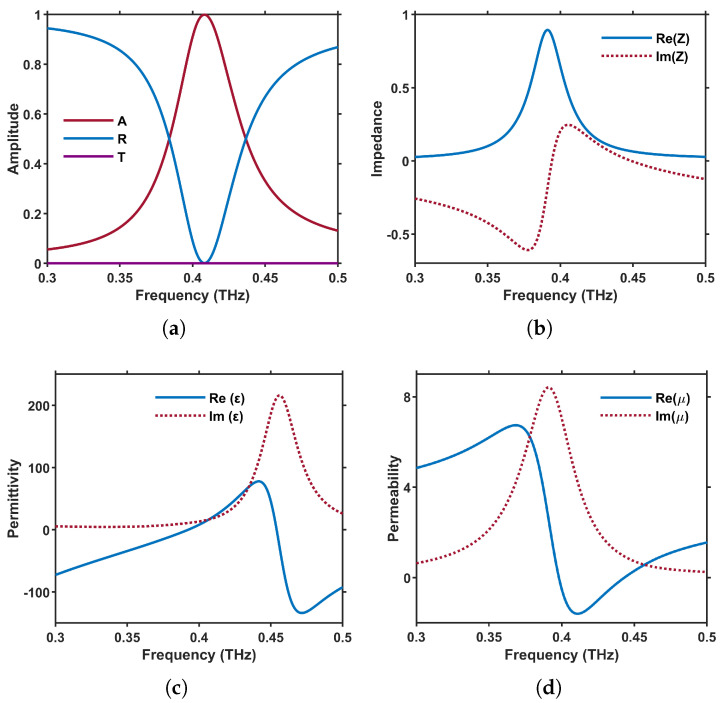
(**a**) Spectral curves of absorbance [A], reflectance [R], and transmittance [T], (**b**) real and imaginary part of the effective surface impedance, (**c**) real and imaginary part of the equivalent permittivity, (**d**) real and imaginary part of the equivalent permeability.

Figure 2a illustrates the absorptance, reflectance, and transmittance spectra of the meta-atom in Figure 1. A peak value of 100% absorptance is achieved at 0.408 THz under normal incidence, confirming the highly efficient absorption performance.

Figure 2b shows the real and imaginary parts of the relative impedance retrieved via (6). The real part approaches 1, and the imaginary part approaches 0 at a frequency close to 0.408 THz, verifying that perfect absorption is obtained when the metasurface impedance matches that of free space.

Moreover, as a metasurface device, it is essential to analyze the equivalent permittivity and permeability. Figure 2c,d depict the retrieved real and imaginary parts of these constitutive parameters. The real part of the effective permeability remains strictly positive across the band, whereas the real part of the effective permittivity crosses the zero line at the absorption peak of 0.408 THz, signaling the excitation of an electric resonance.

The calculated electric resonance shown in Figure 2c is produced by the square InSb patch and appears slightly above the absorption peak, at 0.45 THz. Because of strong near-field coupling between the InSb patch and the underlying continuous InSb film, the induced surface currents flow in opposite directions on these two conductors. This current loop gives rise to the magnetic resonance observed at 0.39 THz in Figure 2d. Such magnetic resonance contributes to achieving optimal impedance matching at the target frequency, thereby minimizing reflection and enabling the metasurface to exhibit nearly perfect absorption at that frequency.

From Figure 2a, we draw that the central working wavelength of the absorptance is approximately 750 μm, with a wavelength-to-period ratio of 4. In the framework of macroscopic electromagnetics and effective medium theory, it is reasonable to approximate the designed system as an isotropic uniform medium when InSb is in its metallic state.

**Figure 3:**
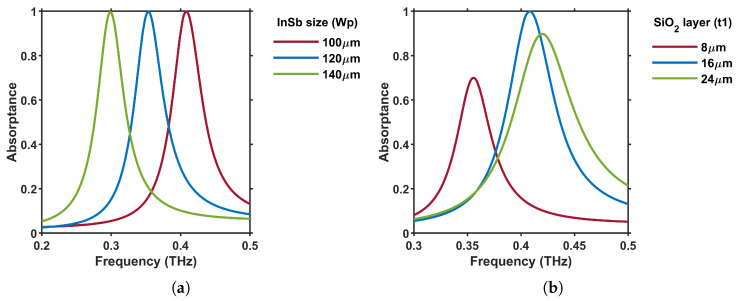
Absorptance spectra of the metasurface in Figure 1 for $\theta^{i}=0^{\circ }$ and TM polarization at $\phi =0^{\circ }$ different values of InSb square size ($W_{p}$) and SiO_2_ thickness ($t_{1}$). (**a**) Effect of $W_{p}=\left\{100,120,140\right\}$μm, and (**b**) $t_{1}=\left\{8,16,24\right\}$μm on the absorptance. All other parameters from Figure 1 remain unchanged.

The absorptance peak of the metasurface can be tuned by varying $W_{p}$ and $t_{1}$ from Figure 1. Figure 3a illustrates the absorptance spectra for $W_{p}=\{$100, 120, 140} μm, showing a redshift in the resonant frequency as $W_{p}$ increases, while the magnitude of the peak absorptance remains unchanged. Similarly, for $t_{1}=\{$8, 16, 24} μm, Figure 3b reveals that the absorptance peak undergoes a blue shift—i.e., it moves to higher frequencies—as the SiO_2_ spacer thickness $t_{1}$ increases, and the peak magnitude changes accordingly. The absorption peak reaches its maximum at 16 μm, suggesting an optimal $t_{1}$ for improved absorption performance.

**Figure 4:**
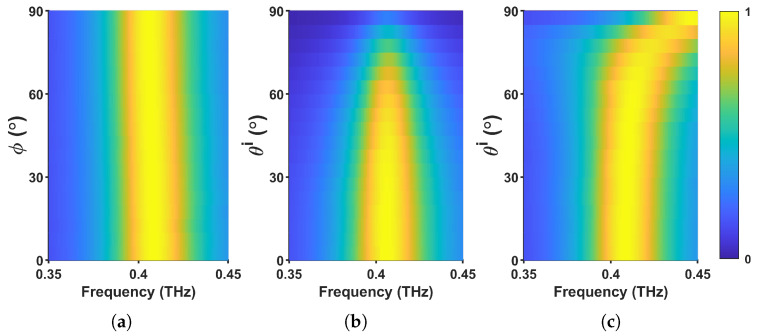
Absorptance spectra of the meta-atom in Figure 1. (**a**) for $\phi ={\left[0,90\right]}^{\circ }$ and $\theta^{i}=0^{\circ }$, demonstrating polarization insensitivity, (**b**) for $\theta^{i}={\left[0,90\right]}^{\circ }$ when the incident wave is TE-polarized, and (**c**) for $\theta^{i}={\left[0,90\right]}^{\circ }$ when the incident wave is TM-polarized.

The performance of the absorber under different polarization states and oblique incidence angles is crucial for practical applications. Figure 4a presents the absorptance as a function of polarization angle under normal incidence. The results indicate that the absorptance remains completely insensitive to polarization variations, which is attributed to the rotational symmetry of the metasurface structure. This polarization-independent behavior makes the design highly suitable for applications requiring robust absorption under arbitrary polarization states.

Beyond normal incidence, it is essential to evaluate the metasurface’s absorption efficiency under oblique incidence. Figure 4b,c illustrate the absorptance spectra for transverse electric (TE) and transverse magnetic (TM) polarized waves as functions of incident angle and frequency. The yellow regions correspond to high absorptance values. In the case of TE-polarized waves (Figure 4b), the absorptance remains stable up to an incident angle of 45°. For angles exceeding 45°, the absorption intensity decreases, and the bandwidth narrows, primarily due to the reduction of the parallel component of the magnetic field as the incident angle increases.

For TM-polarized waves (Figure 4c), the absorptance achieves maximum efficiency at the resonant frequency for incident angles between 0° and 75°. This angle-insensitive performance arises from the strong coupling between localized surface plasmons and the incident wave. The superior absorption stability under TM polarization highlights the metasurface’s robustness in applications involving varying angles of incidence.

### 3.2. Metasurface Polarization Converter

**Figure 5:**
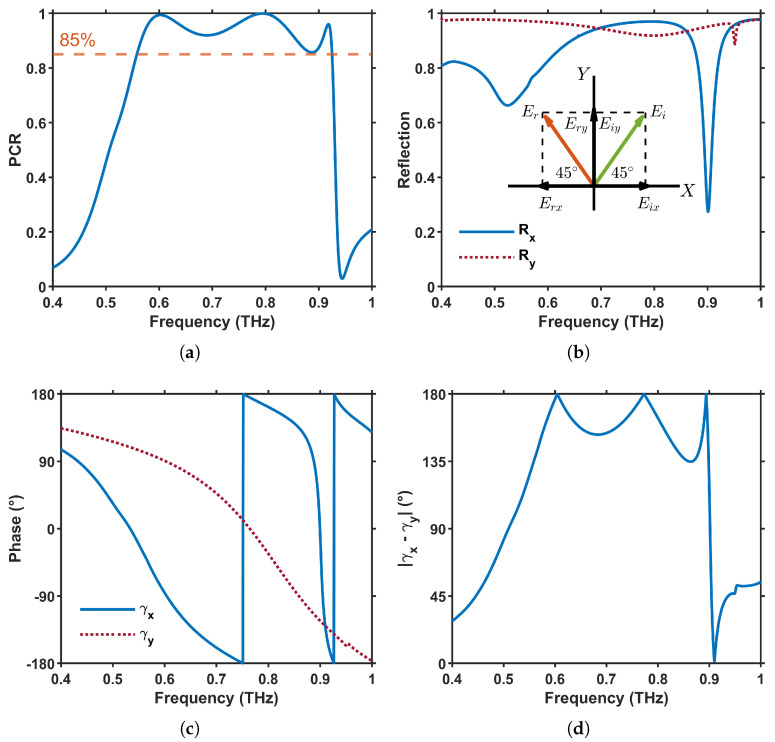
Polarization-conversion performance and reflection characteristics of the meta-atom. (**a**) Simulated polarization-conversion ratio (PCR) for an incident polarization of $\phi =45^{\circ }$. (**b**) Magnitude of the reflection coefficients for the *x*- and *y*-polarized components; the inset sketches the decomposition of the incident and reflected fields into the Cartesian basis. (**c**) Corresponding phases $\gamma_{x}$ and $\gamma_{y}$ of $R_{x}$ and $R_{y}$. (**d**) Phase difference $|\gamma_{x}-\gamma_{y}|$, which remains close to 180° across the high-efficiency band, confirming the condition for robust cross-polarization conversion.

When InSb is in its dielectric state at 200 K, the proposed metasurface operates as a reflective linear-to-linear polarization converter. The physical description begins by expressing the incident and reflected electric fields under normal incidence and decomposing each into its *x*- and *y*-components, as illustrated in the inset of Figure 5b.

(7) $$E_{i}=\hat{x}\;E_{ix}e^{i\delta }+\hat{y}\;E_{iy}e^{i\delta },\;E_{r}=\hat{x}\;R_{x}E_{ix}e^{i\delta }+\hat{y}\;R_{y}E_{iy}e^{i\delta }$$

where $R_{x}=\left|R_{x}\right|e^{i\gamma_{x}}=E_{rx}/E_{ix}$ and $R_{y}=\left|R_{y}\right|e^{i\gamma_{y}}=E_{ry}/E_{iy}$ are the complex reflection coefficients and $\gamma_{x,y}$ their respective phases.

Particular case $\phi =45^{\circ }$:

I confirm. When the incident wave is linearly polarized at this angle, its electric field is equally projected onto *x*- and *y*-axes, resulting in $E_{ix}=E_{iy}=E_{0}/\sqrt{2}$, so that

(8) $$E_{i}=\frac{E_{0}}{\sqrt{2}}\left(\hat{x}+\hat{y}\right),\;E_{r}=\frac{E_{0}}{\sqrt{2}}\left(\hat{x}\;R_{x}+\hat{y}\;R_{y}\right)$$

To analyze the polarization state, we introduce the rotated basis ${\hat{e}}_{\|}=\left(\hat{x}+\hat{y}\right)/\sqrt{2}$, ${\hat{e}}_{⊥}=\left(-\hat{x}+\hat{y}\right)/\sqrt{2}$, which are parallel and orthogonal, respectively, to the incident polarization. By projecting (8) onto these axes, we obtain:

(9) $$E_{\|}^{\;r}=\frac{E_{0}}{2}\left(R_{x}+R_{y}\right),\;E_{⊥}^{\;r}=\frac{E_{0}}{2}\left(R_{y}-R_{x}\right)$$

where $E_{\|}^{\;i}=E_{0}$ is the reference amplitude of the incident field in the same direction.

Polarization–conversion ratio:

The performance of a reflective linear-to-linear converter is quantified. through the polarization–conversion ratio (PCR) [50,51,52,53,54]:

(10) $$\mathrm{PCR}=\frac{|r_{\mathrm{cross}}{|}^{2}}{|r_{\mathrm{cross}}{|}^{2}+{\left|r_{\mathrm{co}}\right|}^{2}}$$

with

(11) $$r_{\mathrm{co}}=\frac{E_{\|}^{\;r}}{E_{\|}^{\;i}}=\frac{R_{x}+R_{y}}{2},\;r_{\mathrm{cross}}=\frac{E_{⊥}^{\;r}}{E_{\|}^{\;i}}=\frac{R_{y}-R_{x}}{2}$$

Equation (11) link the co- and cross-polarized reflection coefficients directly to the Cartesian reflection components ($R_{x},R_{y}$) provided by the full-wave simulation, enabling an immediate evaluation of the PCR. Efficient polarization conversion requires (i) nearly equal reflection magnitudes, $|R_{x}\left|≃\right|R_{y}|\approx 1$, and (ii) a phase difference close to π, $|\gamma_{x}-\gamma_{y}|\approx 180^{\circ }$. Both criteria are simultaneously satisfied within the 0.56 THz to 0.93 THz band, leading to the high PCR values exceeding 85% reported in Figure 5a and corroborated by the magnitude (Figure 5b) and phase (Figure 5c,d) spectra.

The steeper phase variation of $\gamma_{x}$ in Figure 5c arises from the metasurface’s anisotropic design. The horizontal bar in Figure 1b forms a resonant LC path under $E_{x}$ excitation, producing sharp, high-*Q* phase shifts. In contrast, the vertical segment excited by $E_{y}$ is off-resonant in the same band, leading to a smoother, low-*Q* response. This asymmetry enables the strong phase contrast needed for effective polarization conversion.

Because the metasurface is reciprocal and exhibits a strongly anisotropic response, the same polarization–conversion mechanism holds when the incident wave is polarized at the orthogonal angle $\phi =135^{\circ }$. In that case the incident field is aligned with ${\hat{e}}_{⊥}$; after reflection it experiences the same $90^{\circ }$ rotation and emerges with a polarization of $\phi =45^{\circ }$.

**Figure 6:**
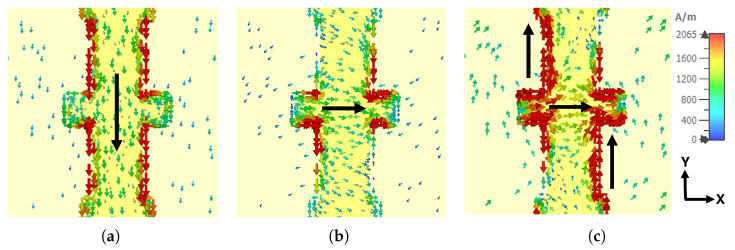
Surface current distribution at three distinct resonance frequencies. The black arrows indicate the dominant direction of the surface current flow at each resonance. (**a**) 0.60 THz, (**b**) 0.79 THz, and (**c**) 0.92 THz.

The analysis of surface current distributions offers valuable insight into the physical mechanism responsible for polarization conversion. Figure 6 reveals how distinct resonant modes are excited as the frequency sweeps through the three peaks identified in Figure 5a. At 0.60 THz (Figure 6a), the induced surface current flows predominantly along the vertical sections of the resonator owing to the strong coupling with the $E_{y}$ component of the incident field; the parallel current configuration between the top resonator and the bottom ground plane thus gives rise to an electric-type resonance within the dielectric spacer. At 0.79 THz (Figure 6b), the current realigns horizontally across the central region, driven mainly by the $E_{x}$ component, and the resulting anti-parallel arrangement produces a magnetic-type resonance via circulating currents in the dielectric. Finally, at 0.92 THz (Figure 6c), strong vertical and horizontal current components coexist, evidencing the simultaneous excitation of both electric and magnetic resonances. These distinct current distributions confirm that the metasurface supports both electric and magnetic resonances across the operating band, contributing to the broadband polarization conversion observed in the device.

**Figure 7:**
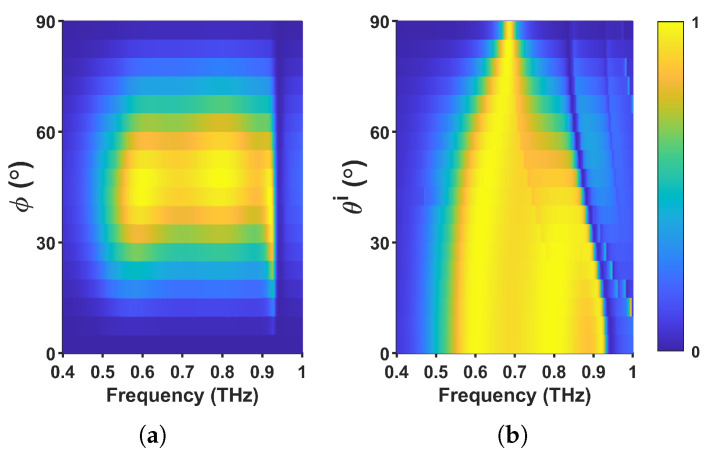
Cross-polarization reflectance as a function of polarization and incident angles. (**a**) Dependence of cross-polarization reflectance on polarization angle and frequency under normal incidence, demonstrating maximum conversion at $45^{{}^{\circ}}$. (**b**) Effect of incident angle on cross-polarization reflectance, showing stable performance up to 45° before narrowing at higher angles.

The dependence of the polarization conversion performance on both polarization and incident angles was also analyzed. Figure 7a presents the cross-polarization reflectance as a function of the polarization angle and frequency under normal incidence. The response of the metasurface is inherently anisotropic due to the geometrical asymmetry of the gold resonator in the *x*–*y* plane, as shown in Figure 1b, which interacts differently with $E_{x}$ and $E_{y}$ as a result of its unequal dimensions along both axes. When the polarization angle is 0° or 90°, the cross-polarization reflectance is nearly zero because the incident *E* and *H* fields align with the coordinate axes, preventing coupling to the anisotropic resonator. As the polarization angle deviates from 0° or 90°, non-zero cross-polarized reflectance appears, reaching a maximum at $45^{\circ }$ where equal *x*- and *y*-components excite the metasurface simultaneously. This confirms that the engineered asymmetry of the gold resonator is the primary driver of efficient polarization conversion.

For practical applications, the polarization converter should maintain stable performance over a range of incident angles. As illustrated in Figure 7b, the intensity and bandwidth of cross-polarization reflectance remain stable up to an incident angle of 45°. This behavior can be attributed to the ratio of the operational wavelength (∼322 μm at 0.93 THz) to the period of the metasurface ( 160 μm), which is approximately 2. This characteristic allows the metasurface to effectively suppress scattering lobes within the working frequency range. However, for incident angles exceeding 45°, the bandwidth of cross-polarization reflectance narrows progressively and eventually converges to a single frequency. The observed insensitivity to incident angles up to 45° highlights the potential of the proposed metasurface for various practical applications where robust polarization conversion is required under different incidence conditions.

### 3.3. Thermal Robustness

**Figure 8:**
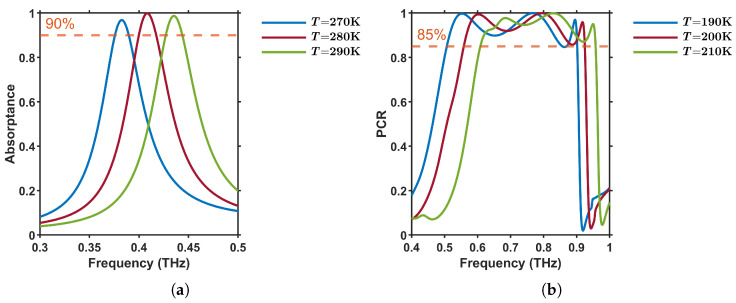
Thermal stability of the metasurface. (**a**) Absorptance spectra at T=[270,280,290] K. (**b**) Polarization conversion ratio (PCR) for T=[190,200,210] K.

To quantify thermal robustness, we recalculated the metasurface response using temperature-dependent plasma frequency $\omega_{p}\left(T\right)$ extracted from the Drude parameters of InSb [37]. Figure 8a shows that the peak absorptance remains above 90% as the temperature varies from 270 K to 290 K, with the resonance center shifting by only 0.052 THz (approximately 12%) relative to its optimal value at 280 K. Likewise, the polarization-conversion ratio plotted in Figure 8b stays above the 85% threshold over a wide frequency range for all three temperatures (190–210 K). The optimal performance is achieved at 200 K, where the PCR remains above 85% from 0.56 to 0.93 THz. At 190 K and 210 K, the corresponding ranges are approximately 0.51–0.90 THz and 0.61–0.95 THz, respectively. These results confirm robust thermal performance.

### 3.4. Comparative Analysis with State-of-the-Art Metasurfaces

Table 1 presents a comparative overview of recent tunable THz metasurfaces in terms of their tuning mechanisms, functionalities, performance metrics, polarization and angular robustness, and technical novelty. Our thermally tunable InSb-based metasurface uniquely combines perfect absorption and efficient polarization conversion within the same unit cell, achieving 100% absorption at 0.408 THz and maintaining PCR>85% over a wide bandwidth (0.56–0.93 THz).

## 4. Conclusions

The study presented a thermally tunable metasurface based on indium antimonide (InSb), designed to operate in the terahertz range with dual functionalities of absorption and polarization conversion. The tunability was enabled by the temperature-dependent permittivity of InSb, which transitions from an insulating state at 200 K to a metallic state at 280 K. This property allowed the metasurface to achieve near-unity absorptance at 0.408 THz under normal incidence while maintaining polarization insensitivity due to its rotational symmetry.

Additionally, the metasurface demonstrated efficient polarization conversion, with a polarization conversion ratio exceeding 85% within the operational bandwidth, [0.56, 0.93] THz. This effect was attributed to the anisotropic response of the structure, leading to a phase difference of approximately 180° between orthogonal reflected components. The polarization conversion remained stable for incident angles up to 45°, ensuring robustness across varying incidence conditions.

In addition, the proposed metasurface demonstrates remarkable thermal robustness, maintaining high absorption and polarization-conversion efficiency over the entire temperature range studied. This stability confirms that the device can operate reliably under typical temperature variations without requiring active tuning.

The obtained results highlight the potential of the proposed InSb-based metasurface for applications in terahertz sensing, polarization manipulation, and wavefront engineering.

## Figures and Tables

**Table 1:** Comparison of recent tunable THz metasurfaces. A = absorptance; PCR = polarization-conversion ratio.

Work (Year)	Active Material/Tuning	Function(s)	Key Performance	Incidence/Pol. Robustness	Novel Advance
This work	InSb, thermal (200 K ↔ 280 K)	Perfect absorber + pol. converter (same pixel)	0.408 THz, A = 100%; 0.56–0.93 THz, PCR > 85%	TE ≤ 45°, TM ≤ 75° for A and ≤45° for PCR	First InSb metasurface combining dual modes; widest temp-tuned PCR band; 100% A
Afra et al., 2025 [55]	VO_2_, thermal	Wide-band absorber/reflector	1.27–2.64 THz, A > 90%	Pol. & angle insensitive	Broadest VO_2_ absorption band (metal phase)
Song et al., 2024 [56]	Graphene, DC bias	Electrically reconfig. pol. converter	240 GHz, ellipticity tunable −0.94 → −0.5 (0–12 V)	(not reported)	First experimentally verified graphene–gold bilayer converter with <12 V drive
Cheng et al., 2021 [57]	InSb (passive)	Narrow-band absorber	1.757 THz, A = 99.9%, Q≈53	-	Ultra-high-Q sensing pixel
Coman et al., 2022 [29]	Cu–polyimide FSRR, geometrical	Variable attenuator	2–5 THz, 10–25 dB loss	-	Broadband THz attenuation window
Xu et al., 2024 [58]	GST225, phase-change	Switchable SP coupler / lens	0.9–1.2 THz (exp.)	-	Non-volatile dual SP device

## Data Availability

The original contributions presented in this study are included in the article. Further inquiries can be directed to the corresponding author.
